# Antibiotic Prescription for the Prevention of Postoperative Complications After Third-Molar Extractions: A Systematic Review

**DOI:** 10.3390/dj13030107

**Published:** 2025-02-28

**Authors:** Nicola De Angelis, Lorenzo Denegri, Ioana Cristina Miron, Catherine Yumang, Paolo Pesce, Domenico Baldi, Francesca Delucchi, Francesco Bagnasco, Maria Menini

**Affiliations:** 1 Department of Surgical Sciences and Integrated Diagnostics (DISC), University of Genoa, 16121 Genoa, Italy; s4487331@studenti.unige.it (L.D.); paolo.pesce@unige.it (P.P.); domenico.baldi@unige.it (D.B.); francesca.delucchi@edu.unige.it (F.D.); francesco.bagnasco@edu.unige.it (F.B.); maria.menini@unige.it (M.M.); 2 Private Practice, 010001 Bucharest, Romania; dr.miron.ioana@gmail.com; 3 Private Practice, 15011 Acqui Terme, Italy; drcatyumang06@gmail.com

**Keywords:** third molar, extraction, dry socket, antibiotic therapy, antibiotic prophylaxis

## Abstract

Background: Third-molar extractions are common procedures often complicated by infections and alveolitis. The use of antibiotics as prophylaxis to prevent these complications is debated due to potential risks and side effects. Therefore, the aim of the present systematic review was to determine the efficacy of antibiotic prescription for the prevention of these complications. Methods: A comprehensive literature search was conducted in MEDLINE/PubMed, Cochrane, and SCOPUS databases up until June 2024. The focused question was “Does the antibiotic prescription influence the incidence of postoperative complications following third-molar extractions in healthy patients?” Systematic reviews assessing complications after third-molar extractions were included. Results: A total of 16 studies were included, revealing that antibiotic use significantly reduces infection risk and dry socket incidence compared to no prescription. Amoxicillin–clavulanic acid was particularly effective. Conclusions: Antibiotics, especially amoxicillin–clavulanic acid, are effective in preventing postoperative infections and alveolitis after third-molar extraction. However, their administration should be carefully considered to balance benefits against potential risks. Evidence supports the judicious use of antibiotics in dental surgery to optimize patient outcomes, minimizing possible adverse effects and the risk of developing antibiotic resistance.

## 1. Introduction

The third molars are the last permanent teeth to erupt in the dental arch and often do not find enough space for their physiological eruption and remain partially or completely impacted [1]. The condition described above is summarized under the name of third-molar impaction. In most cases, problems arising from the misplacement of third molars can only be definitively resolved through surgical extraction. Furthermore, the surgical removal of a third molar becomes necessary in order to prevent the development of chronic inflammation or the formation of osteolytic lesions directly related to the remaining impacted tooth [2,3,4,5]

The risk of experiencing the previously mentioned issues appears to be greater than the infectious risk and complications that may arise from the surgical procedure of avulsion, leading to a preference for the latter option [6,7]. For this reason, the surgical extraction of third molars due to impaction is a widely practiced procedure in dentistry. As in any surgical procedure, the patient is exposed to a risk of immediate and delayed bacterial infection [8,9]. The infectious risk is directly related to several factors, such as the patient’s age, overall health status, unhealthy habits, the incision of the flap and its extension, the type of surgical technique used, the duration of the surgery, and the quality and feasibility of flap suturing. It is the clinician’s task to use the best therapy and surgical technique to minimize the risk of infection [10].

Dental extraction, especially in the case of impacted teeth, leads to a complex sequence of local modifications affecting hard and soft tissues, and the healing of the post-extraction socket can often present complications, one of which is dry socket.

Dry socket is a postoperative issue also known as alveolar osteitis, fibrinolytic alveolitis, septic socket, localized osteomyelitis, or necrotic socket [11,12]. It usually occurs 1 to 3 days after dental extraction and manifests as intense pain in the area where the extraction was performed. It is a complication that can occur with an overall incidence rate of 1–4% after the extraction of any dental element. The probability of developing dry socket is 10 times higher for the extraction of lower teeth compared to upper teeth. In particular, for the mandibular third molars, the incidence is 45% [13,14,15].

In recent years, the debate on the effectiveness of administering antibiotic prophylaxis to patients to prevent the onset of alveolitis following the extraction of mandibular third molars has been quite intense. Indeed, it is now known that taking antibiotics carries risks and possible adverse effects that should not be underestimated, which, in some cases, can even exceed those caused by post-extraction alveolitis [16]. In addition, the worldwide increasing concern regarding the risk of developing antibiotic resistance makes the topic particularly up to date, and antibiotic prescription should be avoided if not considered necessary.

Therefore, the aim of this systematic review is to highlight whether there is a real effectiveness of antibiotic prescription in preventing post-extraction alveolitis following the extraction of impacted mandibular third molars in order to indicate the best clinical protocol to follow in a widely practiced dental surgery intervention to minimize the risk of post-extraction complications and rely on evidence-based medicine for prescribing antibiotics when necessary.

## 2. Materials and Methods

The present systematic review is hereby reported in accordance with the guidelines of the Transparent Reporting of Systematic Reviews and Meta-analyses.

### 2.1. Focused Question

This review aimed to analyze and compare the outcomes of third-molar extractions performed with and without antibiotic prescriptions, as well as different antibiotic regimens. Specifically, it assessed the incidence of postoperative complications.

The focused question was set according to the PICO (population or problem (P), intervention (I), comparison (C), and outcome (O)) strategy, as follows:

- Population: healthy patients with at least one third molar to be extracted;
- Intervention: antibiotic prescription for the extraction of at least one third molar;
- Comparison: another antibiotic prescription or no prescription or placebo for the surgical extraction of at least one third molar;
- Outcomes: alveolitis, infection, swelling, and trismus.

The focused questions were the following:

- Does the antibiotic prescription influence the incidence of postoperative complications following third-molar extractions in healthy patients?
- Which antibiotic prescription is more effective in preventing complications after third-molar extractions in healthy patients?

### 2.2. Search Strategy

A comprehensive literature search was conducted to identify relevant systematic reviews published in English up until June 2024, using databases such as MEDLINE/PubMed, the Cochrane Database of Systematic Reviews, and SCOPUS. The search utilized key terms ((“anti bacterial agents”[Pharmacological Action] OR “anti bacterial agents”[MeSH Terms] OR (“anti bacterial”[All Fields] AND “agents”[All Fields]) OR “anti bacterial agents”[All Fields] OR “antibiotic”[All Fields] OR “antibiotics”[All Fields] OR “antibiotic s”[All Fields] OR “antibiotical”[All Fields]) AND ((“molar, third”[MeSH Terms] OR (“molar”[All Fields] AND “third”[All Fields]) OR “third molar”[All Fields] OR (“wisdom”[All Fields] AND “tooth”[All Fields]) OR “wisdom tooth”[All Fields]) AND (“extract”[All Fields] OR “extract s”[All Fields] OR “extractabilities”[All Fields] OR “extractability”[All Fields] OR “extractable”[All Fields] OR “extractables”[All Fields] OR “extractant”[All Fields] OR “extractants”[All Fields] OR “extracted”[All Fields] OR “extractibility”[All Fields] OR “extractible”[All Fields] OR “extracting”[All Fields] OR “extraction”[All Fields] OR “extractions”[All Fields] OR “extractive”[All Fields] OR “extractives”[All Fields] OR “extracts”[All Fields]))) AND meta-analysis[Filter] OR systematicreview[Filter]) combined with Boolean operators (“AND”/”OR”) to ensure a thorough examination of the available literature.

**Figure 1:**
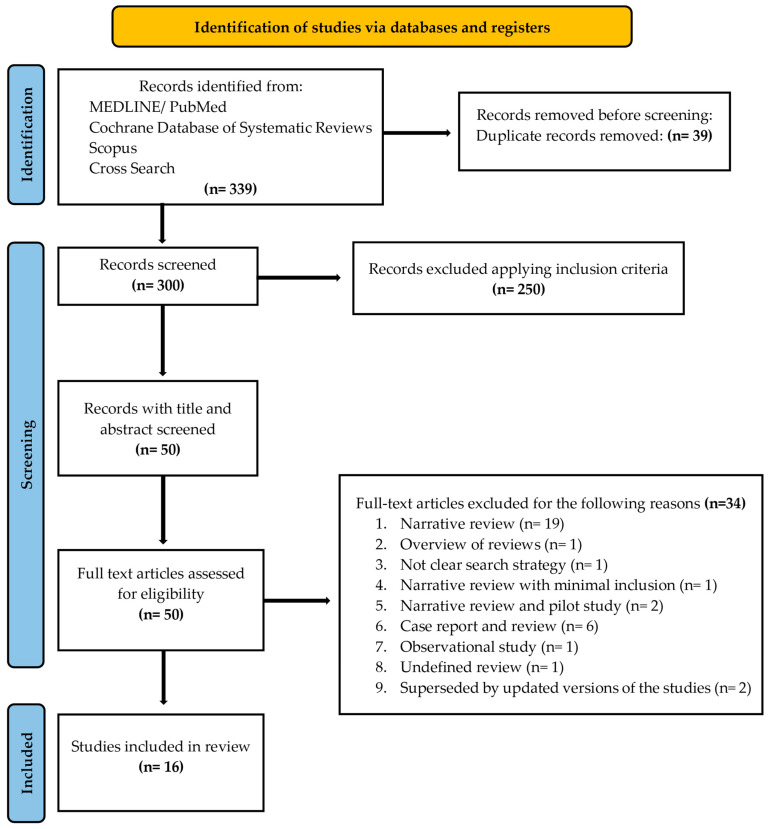
PRISMA 2020 flow diagram for new systematic reviews, including searches of databases and registers only. Search has been conducted by reading full texts of all the records, before judging the exclusion.

A PRISMA flow diagram (Figure 1) was included to clearly outline the search methodology and selection process, adhering to best practices for systematic reviews. We hand-searched the content pages of the most relevant journals in the field. In addition, the search was complemented by manual searches of the reference lists of all the systematic reviews captured.

### 2.3. Inclusion and Exclusion Criteria

The criteria for study inclusion were as follows:

- Systematic reviews of clinical studies evaluating the analyzed outcomes.

The exclusion criteria were as follows:

- Narrative reviews;
- Systematic reviews of in vitro studies;
- Systematic reviews of animal studies;
- Systematic reviews of systematic reviews;
- Methodology unclear, whereby the search strategy and/or the inclusion and exclusion criteria had not been clearly defined.

No restrictions related to language nor the year of publication were imposed. Studies not meeting the inclusion criteria were excluded.

### 2.4. Screening Method

Two reviewers (N.D.A. and C.S.Y.) independently performed the primary search and, afterwards, the screening of the titles and abstracts was performed manually. The full texts of potentially eligible reviews were obtained and independently assessed by the two reviewers to reach a decision and select the studies that met the inclusion criteria. Any discrepancy was resolved through a discussion with a third author (M.M.).

### 2.5. Data Extraction

Data were extracted independently by two reviewers (N.D.A. and C.S.Y.) using an Excel spreadsheet (Microsoft, Redmond, WA, USA) specifically created for this review. The data extracted included the title, authors, year of publication, type of study (e.g., systematic review or metanalysis), focused question of the study clearly reported, antibiotic regimen clearly mentioned, number of studies included, years in which the studies in the systematic reviews were published, total number of patients reported, total number of extractions, and outcomes.

During this process, any discrepancy was resolved through a consensus discussion with a third author (M.M.).

### 2.6. Quality Assessment

The AMSTAR 2 (a measurement tool to assess systematic reviews), as suggested by Shea BJ et al. [17], was used to assess the methodological quality of the systematic reviews included in the present research.

## 3. Results

### 3.1. Inclusion and Exclusion of Articles

A flow diagram reporting the screening and selection of studies is presented in Figure 1. The electronic search identified 339 papers in total, and after duplicates’ removal, 300 studies remained. Applying the inclusion criteria, 50 articles were screened. After manual screening of the title and abstract and full text, 34 studies were excluded, and the full-text of 16 articles was analyzed. Table 1 reports the excluded studies and the reasons for the exclusion.

### 3.2. Description of Selected Articles

Sixteen studies published between 2012 and 2024 were included (Table 2), and they consisted of nine systematic reviews and meta-analyses (Isiordia-Espinoza et al. (2015) [46], Arteagoitia et al. (2016) [47], Ramos et al. (2016) [48], Menon et al. (2019) [49], Arteagoitia I et al. (2022) [50], Falci et al. (2022) [51], Wang et al. (2022) [52], Torof et al. (2023) [53], Camps-Font et al. (2024) [54]) and seven systematic reviews (Marcussen et al. (2016) [55], Marchionni et al. (2017) [56], Cervino et al. (2019) [57], Daly et al. (2021) [58], Milic et al. (2021) [59], Lodi et al. (2021) [60], and Sologova et al. (2022) [61]). 

Eight reviews reported the comparison of antibiotic therapy versus a placebo, while two studies reported a comparison between different antibiotics administered before and after the surgical extraction of lower third molars. One study focused on the effect of clindamycin, without comparisons, in reducing postoperative infections. The others described the efficacy of antibiotic prescription for post-surgical complications without clear comparisons between different antibiotics.

### 3.3. Quality Assessment of the Included Studies

A quality assessment of the 16 systematic reviews selected is reported in Table 3 and Table 4.

### 3.4. Which Antibiotic Is the Most Effective in Preventing Postoperative Complications?

**Figure 2:**
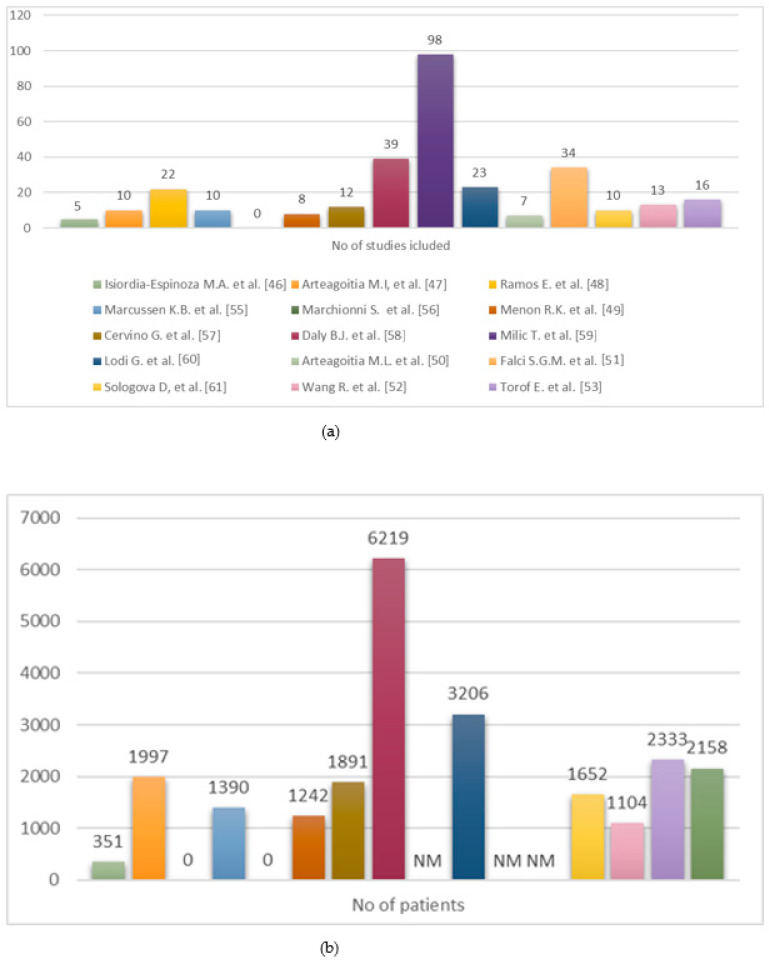
(**a**) Distribution of included RCTs across systematic reviews. (**b**) Number of patients enrolled in included RCTs [46,47,48,49,50,51,52,53,55,56,57,58,59,60,61].

All sixteen included articles agreed about the role of antibiotics in the prevention of postoperative complications, evaluating different prescriptions (Figure 2).

Camps-Font et al. (2024), in their systematic review [54], evaluated the effectiveness of amoxicillin combined with clavulanic acid, amoxicillin, metronidazole, azithromycin, and clindamycin compared to a placebo or no-treatment group across 16 included RCTs. The meta-analysis of direct comparisons showed that antibiotics were significantly more effective than a placebo or no treatment in reducing surgical-site infections (OR 0.36, 95% CI 0.22–0.57, *p* < 0.001, I^2^ = 4%), with a number needed to treat (NNT) of 18. The network meta-analysis (NMA) also demonstrated a significant advantage of the pre- and postoperative use of amoxicillin plus clavulanic acid compared to a placebo or no treatment, with a low inconsistency in the network (χ^2^ = 6.75; *p* = 0.239).

**Figure 3:**
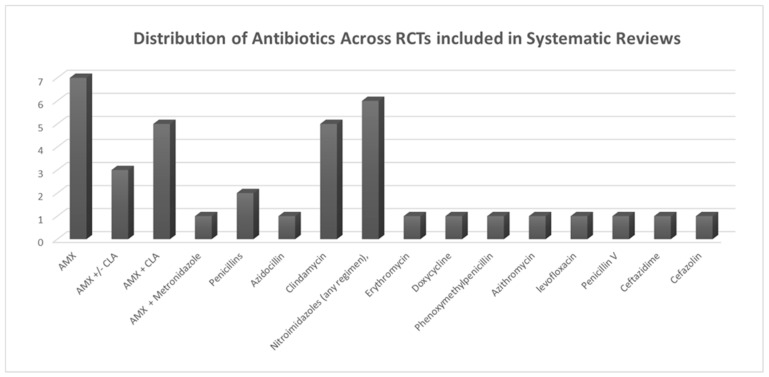
Distribution of antibiotic usage across RCTs included in systematic reviews.

Falci et al. (2022) [51] examined the effectiveness of antibiotics compared to a placebo in preventing surgical-site infections (including alveolar osteitis, commonly referred to as dry socket) following third-molar surgery. Their systematic review included 34 RCTs. The findings indicated that patients treated with amoxicillin experienced a 44% reduction in their relative risk of infection (RR 0.56, 95% CI 0.38–0.84; low quality of evidence). Similarly, patients treated with metronidazole achieved a 49% relative risk reduction (RR 0.51, 95% CI 0.31–0.84; low quality of evidence). Based on the SUCRA rankings, metronidazole emerged as the top antibiotic for infection prevention (75.1%), with amoxicillin ranking second (66.5%) (Figure 3).

Ramos et al. (2016) [48] evaluated the role of systemic antibiotics in preventing infections after third-molar surgery, restricting their analysis to randomized, double-blind, placebo-controlled trials. A total of 21 studies were included in their systematic review. The antibiotics analyzed included penicillin and nitroimidazoles. The evidence suggested that antibiotic administration to prevent infections post third-molar extraction reduced the infection risk by 57%.

In terms of dry socket rates, notable differences were identified. Patients treated with nitroimidazoles had a significantly higher rate of dry socket (7.22%) compared to those treated with penicillin (3.05%). However, this trend was also observed in the control groups, where the infection rate was 8.48% for studies involving penicillin and 16.55% for studies involving nitroimidazoles.

There is evidence that antibiotics administered to prevent infection after third-molar extraction reduce the risk of infection by 57%.

Regarding the dry socket rate, we found significant differences. The extent was twice as high in patients treated with nitroimidazoles (7.22%) than in those treated with penicillin (3.05%). However, this pattern was also detected in the respective control groups, with the rate of infection being 8.48% in the control groups of the studies using penicillin and 16.55% in those using nitroimidazoles.

Arteagoitia et al. (2016) [47] explored the effectiveness of prophylactic amoxicillin, with or without clavulanic acid, in reducing the incidence of dry socket following third-molar extractions, reviewing data from 10 studies. The relative risks were found to be 0.563 for amoxicillin (95% CI: 0.295 to 1.08, *p* = 0.082) and 0.215 for amoxicillin–clavulanic acid (95% CI: 0.117 to 0.395, *p* < 0.001). The meta-analysis showed that, in the control group, the mean infection rate was 8%, with variability across the included trials. In studies using amoxicillin, the mean infection rate in the placebo group was 5%, compared to 13% in those using amoxicillin–clavulanic acid.

Cervino et al. (2019) [57] reviewed the most commonly employed antibiotic protocols in dentistry, particularly in surgical procedures for impacted third molars, analyzing 12 RCTs that assessed the postoperative infection rates. The results identified penicillin with clavulanate as the most frequently utilized protocol, demonstrating effective clinical and prophylactic outcomes in managing infections. This widely adopted approach appears to offer high predictability and safety. The study aimed to highlight factors supporting or opposing different therapeutic approaches. Current clinician preferences favor amoxicillin, although debate persists in the literature regarding the necessity of antibiotic protocols.

Wang et al. (2022) [52] examined the efficacy of amoxicillin (AMX) and amoxicillin–clavulanic acid (AMX-CLA) in reducing the postoperative infection rates after third-molar surgery. Data from 13 RCTs were included in the meta-analysis, which revealed a statistically significant reduction in infection risk with AMX/AMX-CLA (RR: 0.29, 95% CI: 0.18–0.45; I^2^ = 0%, *p* < 0.00001). Pooled data from over 2000 patients indicated a 71% decrease in the infection risk with these antibiotics. The absence of heterogeneity (I^2^ = 0%) and publication bias in the analysis bolstered the reliability of the findings.

Menon et al. (2019) [49] assessed the effectiveness of amoxicillin and amoxicillin–clavulanic acid in preventing postoperative infections and complications after third-molar surgery, as well as their adverse effects in both patients and healthy volunteers. Of the 11 studies that met the inclusion criteria for qualitative analysis, 8 were included in the meta-analysis. The findings demonstrated that both antibiotics are effective in reducing infection risk after third-molar extraction.

Sologova et al. (2022) [61] reviewed and categorized the use of antibacterial drugs to prevent postoperative complications in outpatient wisdom tooth surgeries, selecting 10 studies based on inclusion and exclusion criteria. Amoxicillin, both alone and in combination with clavulanic acid, was identified as the most commonly used antibiotic in varying dosages and durations.

Arteagoitia I. et al. (2022) [50] investigated the use of clindamycin to prevent infection after oral surgery. Seven studies were included in the qualitative synthesis, with four contributing to the quantitative synthesis (meta-analysis). The findings concluded that there was insufficient evidence to evaluate the effectiveness of clindamycin for oral surgical procedures beyond third-molar extractions. The null hypothesis—stating that clindamycin is not effective in preventing infection in third-molar surgery regardless of dosage—could not be rejected.

Isiordia-Espinoza et al. (2015) [46] assessed the impact of amoxicillin on surgical wound infection risk and adverse effects in healthy individuals undergoing third-molar extractions. Among fifteen relevant studies, five met the inclusion criteria and were evaluated using the Oxford Quality Scale, scoring a minimum of three points. The study concluded that administering amoxicillin before or after surgery did not significantly reduce infection risk compared to a placebo or no treatment. Data from 351 patients across five studies revealed that 198 patients received amoxicillin pre or post surgery, with 3 (2%) developing infections, compared to 11 (7%) among the 153 untreated patients. The absolute risk reduction was 6% (95% CI: 1.24% to 10.11%), with an NNT of 18 (95% CI: 9.9 to 80.5) to prevent one infection. Only one study showed a reduced infection risk, favoring amoxicillin. The pooled meta-analysis results demonstrated no statistically significant infection risk reduction for the amoxicillin groups compared to the untreated or placebo groups.

Lodi et al. (2021) [60] analyzed 23 RCTs investigating preoperative antibiotic use and its impact on infection rates. The results showed that preoperative antibiotics reduced the infection rate by 66%.

Torof et al. (2023) [53] reviewed the variability in clinical practices and the lack of consensus on optimal antibiotic protocols for invasive dental procedures, analyzing 16 RCTs. Antibiotics significantly reduced infection rates (*p* < 0.01). However, no significant impact was found on dry socket prevention (*p* = 0.34), and the NNT values (17–97) suggested limited efficacy in justifying routine use.

Milic et al. (2021) [59] provided evidence-based recommendations for prophylactic antibiotic use in surgical third-molar extractions. Out of 98 reviewed papers, 31 studies evaluated prophylactic antibiotics for third-molar surgery. Recommendations were based on factors such as impaction depth, osteotomy requirements, trauma to surrounding tissues, and postoperative inflammation. However, the optimal timing of antibiotic administration (preoperative, intraoperative, or postoperative) remained unclear.

Marcussen et al. (2016) [55] assessed the effectiveness of a single preoperative antibiotic dose—administered orally, intravenously, intramuscularly, or topically—in preventing infections and alveolar osteitis during lower-third-molar extractions involving osteotomy. Their review included 10 RCTs, and pooled results indicated a significant reduction in alveolar osteitis prevalence (OR = 0.35; 95% CI: 0.13 to 0.96; *p* = 0.04).

Marchionni et al. (2017) [56] explored the benefits and drawbacks of systemic prophylactic antibiotics for tooth extractions other than third molars, compared to no antibiotics or a placebo. No RCTs were found addressing the necessity of antibiotics for non-third-molar extractions. The study emphasized the need for well-designed RCTs to clarify the role of antibiotics in routine tooth extraction.

Daly et al. (2021) [58] investigated local interventions for preventing and treating alveolar osteitis (dry socket) following tooth extractions across 49 trials. This review suggests that chlorhexidine treatments are effective in preventing dry socket, while other interventions, such as Alvogyl or platelet-rich plasma, did not demonstrate significant efficacy

### 3.5. Which Is the Recommended Antibiotic Prescription for the Prevention of Postoperative Complications in Cases of Third-Molar Extraction in Healthy Patients?

Only five out of the sixteen included reviews reveal details about the prescription dosage of antibiotics. (Table 5)

Falci et al. (2022) [51] demonstrated that the postoperative administration of 750 mg amoxicillin, postoperative amoxicillin combined with clavulanate at doses of 500 mg + 125 mg or 2000 mg + 125 mg, and the preoperative administration of 800 mg metronidazole were effective in preventing infections and dry socket compared to the placebo.

Wang et al. (2022) [52] reported that both amoxicillin (AMX) and amoxicillin–clavulanic acid (AMX-CLA) were associated with a significant reduction in infection risk following impacted third-molar surgeries. Their findings highlighted that antibiotic administration, whether preoperative or postoperative, effectively reduced infection rates. They strongly recommended the preoperative administration of amoxicillin or amoxicillin–clavulanic acid for systemic antibiotic prophylaxis when indicated.

Milic et al. (2021) [59] strongly recommended a single 2 g dose of preoperative oral amoxicillin for patients undergoing lower-mandibular-third-molar extractions with osteotomy to reduce the risk of surgical-site infections (SSIs). Similarly, Marcussen et al. (2016) [55] concluded that a single 2 g dose of oral amoxicillin before the surgical extraction of lower third molars with osteotomy significantly decreased the incidence of SSIs. Additionally, they found that a single 0.8 g dose of penicillin V administered preoperatively significantly reduced the occurrence of alveolar osteitis.

Sologova et al. (2022) [61] reviewed antibiotic administration practices in their systematic analysis. Four studies involved administering antibiotics exclusively before surgery, while two studies used antibiotics only postoperatively. In four other studies, preoperative and postoperative antibiotic regimens were compared across different patient groups. Two studies specifically examined amoxicillin administered both before and after surgery, although the antibiotic dosages varied across trials.

## 4. Discussion

The comparative analysis of various antibiotic prescription for preventing postoperative complications, particularly surgical-site infections (SSIs) and dry socket, has provided valuable insights. These findings, derived from numerous randomized controlled trials (RCTs) and synthesized through systematic reviews, offer actionable recommendations for clinical practice while underscoring areas needing further investigation. Amoxicillin and amoxicillin–clavulanic acid consistently emerged as highly effective in reducing postoperative infections. Camps-Font et al. (2024) [54] highlighted the superior efficacy of amoxicillin–clavulanic acid, with an odds ratio (OR) of 0.36 and a number needed to treat (NNT) of 18. These findings align with Wang et al. (2022) [52], who reported a 71% reduced infection risk associated with amoxicillin and amoxicillin–clavulanic acid, supported by low heterogeneity (I^2^ = 0%). This evidence is further reinforced by Arteagoitia et al. (2016) [48], who demonstrated the greater impact of amoxicillin–clavulanic acid (RR 0.215, *p* < 0.001) compared to amoxicillin alone (RR 0.563, *p* = 0.082). These consistent results recommend the use of amoxicillin–clavulanic acid, particularly in high-risk patients undergoing oral surgery. 

The findings on metronidazole were also notable. Falci et al. (2022) [51] observed a 49% relative risk reduction with metronidazole prescription, ranking it as the most effective antibiotic for SSI prevention (SUCRA: 75.1%). This positions metronidazole as a viable alternative, particularly for patients intolerant to penicillin-based antibiotics. Also, clindamycin, often prescribed as a penicillin alternative, showed variable results. However, Arteagoitia I. et al. (2022) [50] found insufficient evidence supporting clindamycin’s efficacy, highlighting the need for further RCTs to clarify its role in postoperative infection prevention.

The prevention of dry socket also showed promising results with antibiotic use. Lodi et al. (2021) [60] reported a reduction in dry socket risk from 6.3% in the placebo group to 3.8% in the antibiotic group, underscoring the potential benefit of prophylactic antibiotics in mitigating this complication. The SUCRA rankings provided by Camps-Font et al. (2024) [54] identified clindamycin (73.7%) and azithromycin (71.3%) as the leading antibiotics for dry socket prevention, although the evidence for clindamycin remains inconclusive based on findings from other studies. For infection prevention, Falci et al. (2022) [51] noted that postoperative amoxicillin–clavulanic acid achieved the highest SUCRA ranking (71.7%). Wang et al. (2022) [52] corroborated this, emphasizing significant risk reductions with both the preoperative (RR 0.41) and postoperative (RR 0.18) administration of amoxicillin or amoxicillin–clavulanic acid. This suggests that timing is critical, with both pre- and postoperative regimens offering protective benefits

The optimal dosage and timing of antibiotic administration remain pivotal in maximizing efficacy while minimizing resistance risks. Falci et al. (2022) [51] recommended postoperative regimens such as 750 mg of amoxicillin or amoxicillin–clavulanate combinations of 500 mg + 125 mg or 2000 mg + 125 mg. Milic et al. (2021) [59] advocated a single preoperative dose of 2 g oral amoxicillin for lower-mandibular-third-molar extractions, significantly reducing the SSI rates. Similarly, Marcussen et al. (2016) [55] highlighted the effectiveness of a single 2 g dose of amoxicillin or 0.8 g of penicillin V in reducing surgical-site infections and alveolar osteitis, respectively. These findings support a shift toward single-dose preoperative regimens, offering simplicity and efficacy while possibly reducing antibiotic side effects. 

While the evidence strongly supports certain antibiotics and regimens, it is essential to consider the multifactorial nature of postoperative complications. Factors such as the tooth’s condition, presence of infection, patient comorbidities, surgical technique, and the surgeon’s learning curve significantly influence outcomes. In particular, patients with systemic conditions such as risk of endocarditis or diabetes require tailored prophylactic strategies, and more complex extractions may necessitate extended antibiotic courses in specific clinical conditions. These contextual factors, though not directly addressed in this review, must be integrated into clinical decision making. This underscores the need for a holistic approach, combining evidence-based antibiotic use with individualized patient assessment. The systematic review of other systematic reviews provides a robust foundation for these recommendations, leveraging data from high-quality RCTs and meta-analyses. The inclusion of multiple systematic reviews enhances the generalizability of findings and reduces bias. 

However, some limitations must be acknowledged. First, the variability in study designs, populations, and antibiotic regimens introduces heterogeneity that may influence pooled estimates. For example, differences in SSI definitions or follow-up durations can affect reported outcomes. Second, while SUCRA rankings provide a comparative framework, their reliance on indirect evidence necessitates cautious interpretation. For instance, the high ranking of clindamycin for dry socket prevention conflicts with direct evidence questioning its efficacy [50]. This highlights the need for head-to-head trials to validate SUCRA-based conclusions. Finally, the review’s scope, focusing primarily on antibiotics, does not account for non-pharmacological measures such as surgical techniques, antiseptic use, or patient education. Future research should explore these complementary strategies, particularly in settings where antibiotic stewardship is a priority.

## 5. Conclusions

The present review of systematic reviews highlights that antibiotic prescription, especially amoxicillin–clavulanic acid, is effective in preventing postoperative infections and alveolitis after third-molar extraction. However, their administration should be carefully considered to balance benefits against potential risks.

In order to bridge critical knowledge gaps, future research should focus on how the usage of antibiotics is affected by patient-specific characteristics, including comorbidities, systemic diseases, and local factors such as bone quality or healing ability. The post-extraction evolution may be greatly impacted by these characteristics, which should be more effectively included into customized treatment plans.

Additionally, the global issue of antibiotic resistance highlights the need for strategies that minimize its development while maintaining therapeutic effectiveness. Exploring alternative or complementary therapies, such as non-antibiotic interventions, could significantly enhance existing treatment approaches. Future studies should prioritize reporting on these methods and their outcomes.

Lastly, therapies that improve postoperative quality of life should also be the subject of study. Promoting sustainable, patient-centered care demands an understanding of how different approaches, such as alternative treatments, may accelerate healing, decrease discomfort, and increase patient satisfaction.

## Figures and Tables

**Table 1:** Reasons for exclusion of 34 articles after reading the full text.

Reason of Exclusion	Excluded Articles
Narrative review (n = 19)	Cho H. et al., 2017 [18]
	Ghosh A. et al., 2022 [13]
	Bouloux GF. et al., 2007 [10]
	Steel BJ. et al., 2022 [19]
	Oomens M. et al., 2012 [20]
	Seymour RA. et al., 2002 [21]
	Murphy S., 2022 [22]
	Piecuch JF., 2012 [23]
	Clauser B. et al., 2009 [24]
	Rodrigues WC. et al., 2015 [25]
	Susarla SM et al., 2011 [26]
	Mehrabi M. et al., 2007 [27]
	Barasch A. et al., 2008 [28]
	Marghalani A., 2014 [29]
	Meechan JG. et al., 1993 [30]
	Xue P. et al., 2015 [31]
	Houston JP. et al., 2002 [14]
	Berenstein D. et al., 2013 [32]
	Sands T. et al.,1993 [33]
	Zeitler DL., 1995 [34]
Overview of reviews (n = 1)	Cao Y. et al., 2023 [35]
No clear search strategy (n = 1)	Fridrich KL. et al., 1990 [16]
Narrative review with minimal inclusion	Fenton DA. et al., 2012 [36]
Narrative review and pilot study (n = 2)	van Eeden SP. et al., 2006 [37] Chapnick P. et al., 1992 [12]
Case report and review (n = 6)	Fukuda M. et al., 2017 [38]
	Wang R. et al., 2014 [39]
	Patel D. et al., 2001 [40]
	Pepato AO. et al., 2012 [41]
	Bertolai R. et al., 2007 [2]
	Morey-Mas M. et al., 1996 [42]
Observational study (n = 1)	Polastri F. et al., 1983 [43]
Undefined review (n = 1)	Oomens MA. et al., 2012 [20]
Superseded by updated versions of the studies (n = 2)	Lodi G. et al., 2012 [44]
	Daly B. et al., 2012 [45]

**Table 2:** Main characteristics of the systematic reviews included in the present review.

Authors	Year of Publication	Design	Design of the Studies Included	No of Studies Included	No of Patients
Isiordia-Espinoza. et al. [46]	2015	Syst. Rev + Metan.	RCTs	5	351
Arteagoitia et al. [47]	2016	Syst. Rev + Metan.	RCTs	10	1997
Ramos et al. [48]	2016	Syst. Rev + Metan.	RCTs Prospective Retrospective	22	NM
Marcussen et al. [55]	2016	Syst. Rev	RCTs	10	1390
Marchionni et al. [56]	2017	Syst. Rev	RCTs	Number of studies not clearly reported	Number of patients not clearly reported
Menon et al. [49]	2019	Syst. Rev + Metan.	RCTs	8	1242
Cervino et al. [57]	2019	Syst. Rev	RCTs	12	1891
Daly et al. [58]	2021	Syst. Rev	RCTs	39	6219
Milic et al. [59]	2021	Syst. Rev	RCTs	98	NM
Lodi et al. [60]	2021	Syst. Rev	RCTs	23	3206
Arteagoitia L. et al. [50]	2022	Syst. Rev + Metan.	RCTs	7	NM
Falci et al. [51]	2022	Syst. Rev + Metan.	RCTs	34	NM
Sologova et al. [61]	2022		RCTs	10	1652
Wang et al. [52]	2022	Syst. Rev + Metan.	RCTs	13	1104
Torof et al. [53]	2023	Syst. Rev + Metan.	RCTs Double-blind and placebo-controlled clinical trial	16	2333
Camps-Font et al. [54]	2024	Syst. Rev + Metan.	RCTs	16	2158

**Table 3:** Aims and prescription compared in included systematic reviews.

Authors	Year of Publication	Design of the Studies Included	Aim	Prescription Compared
**Isiordia-Espinoza et al. [46]**	2015	RCTs	To assess the risk of surgical wound infection and the adverse effects of amoxicillin healthy patients who required excision of third molars.	Antibiotics (amoxicilin) and placebo vs. untreated group.
**Arteagoitia et al.** [47]	2016	RCTs	To assess the efficacy of prophylactic amoxicillin with or without clavulanic acid in reducing the incidence of dry socket and/or infection after third molar extraction.	Amoxicillin vs. amoxicillin + clavulanic acid.
**Ramos et al. [48]**	2016	RCTs Prospective Retrospective	To assess the efficacy of systemic antibiotics in reducing the frequencies of these complications after extraction.	Prophlactic antibiotics vs. placebo groups.
**Marcussen et al. [55]**	2016	RCTs	To evaluate effectiveness of a single dose of preoperative antibiotic administered per-orally, intravenously, intramuscularly or topically for preventing infection and alveolar osteitis in lower third molar surgical extraction implying osteotomy	Prophylactic antibiotics vs. no antibiotics/placebo on infection after surgical removal.
**Marchionni et al. [56]**	2017	RCTs	To assess the beneficial or harmful effects of systemic prophylactic antibiotics at extraction of teeth, apart from third molars, vs. no antibiotic or placebo administration. Furthermore, if antibiotics are beneficial, to determine which type, dosage, duration and timing of administration is the most effective.	Comparing the administration of various prophylactic antibiotic regimens vs. no antibiotics to people undergoing extraction of teeth, not including third molars, were included.
**Menon et al. [49]**	2019	RCTs	The efficacy of amoxicillin/amoxicillin–clavulanic acid for reducing the risk of postoperative infection after third molar surgery and to evaluate the adverse outcomes	Prophlactic antibiotics vs. placebo groups.
**Cervino et al. [57]**	2019	RCTs	To highlight the most widely antibiotic protocols applied to the dental field, especially in the surgical treatment of impacted wisdom teeth	Antibiotics vs. placebo and comparison between different antibiotics protocols
**Daly et al. [58]**	2021	RCTs	To assess the effects of local interventions for the prevention and treatment of alveolar osteitis (dry socket) following tooth extraction.	Comparison local interventions (CHX and intrasocket interventions, platelet rich plasma, acellular dermal matrix patches) and placebo groups
**Milic et al. [59]**	2021	RCTs	To provide evidence-based recommendations for antibiotic prophylaxis. To identify antibiotic regimens for use in oral surgery.	Antibiotics use preoperative, perioperative or postoperative.
**Lodi et al. [60]**	2021	RCTs	To determine the effect of systemic antibiotic prophylaxis on the prevention of infectious complications following tooth extractions.	Antibiotics vs. placebo groups.
**Arteagoitia L. et al. [50]**	2022	RCTs	To determine the effect of clindamycin in the prevention of infection after oral surgery.	Antibiotics (clindamycin) vs. placebo groups.
**Falci et al. [51]**	2022	RCTs	To determine whether antibiotics, compared to placebo, can prevent infection or dry socket after third molar surgery	Antibiotics vs. placebo groups.
**Sologova et al. [61]**	2022	RCTs	To evaluate and systematize the use of antibacterial drugs in order to prevent postoperative	Antibiotics vs. placebo/untreated groups, anti-inflammatory drugs.
**Wang et al. [52]**	2022	RCTs	To assess the efficacy of AMX/AMX-CLA for infection control after third molar surgery.	Antibiotics vs. placebo groups.
**Torof et al. [53]**	2023	RCTs Double-blind and placebo controlled clinical trial	To investigate this disparity of practices and the absence of global and local recent consensus on the most appropriate antibiotic interventions around invasive dental procedures.	Antibiotics vs. placebo or untreated group.
**Camps-Font et al. [54]**	2024	RCTs	To compare the risk of dry socket and surgical site infection after the removal of lower third molars with different prophylactic antibiotics	Amoxicillin (with and without clavulanic acid) vs. metronidazole and azithromycin, or clindamycin against no treatment/placebo group.

**Table 4:** Quality evaluation.

	Study No.	[46]	[47]	[48]	[55]	[56]	[49]	[57]	[58]	[59]	[60]	[50]	[51]	[61]	[52]	[53]	[54]
Criteria																	
**Research question and inclusion criteria PICO**		2	2	1	1	2	1	1	1	1	1	1	2	2	1	1	1
**Protocol registered before commencement of the review**		2	2	1	1	2	1	1	1	1	1	1	2	2	1	1	1
**Explanation of selection of drawings from the included studies**		2	2	1	1	2	1	1	1	1	1	1	2	2	1	1	1
**Adequacy of the literature search**		2	2	1	1	2	1	1	1	1	1	1	2	2	1	1	1
**Duplicate study selection**		2	1	2	2	2	2	1	2	2	1	2	1	2	2	2	1
**Duplicate data extraction**		2	1	2	2	2	2	1	2	2	1	2	1	2	2	2	2
**List and justification of excluded studies**		2	1	2	2	2	2	1	2	2	1	2	1	2	2	2	2
**Studies included described in detail**		3	1	1	3	3	1	1	1	1	1	3	1	3	1	1	2
**Risk of bias from individual studies being included in the review**		1	1	4	1	1	1	1	4	1	1	1	1	1	1	1	2
**Sources of financing of included studies reported in review**		1	1	4	1	1	1	1	4	1	1	1	1	1	1	1	2
**Appropriateness of meta-analytical methods**		1	1	4	1	1	1	1	4	1	1	1	1	1	1	1	2
**If meta-analysis: bias risk of included studies taken into account**		1	1	1	1	1	2	2	1	1	1	1	1	1	1	1	2
**Risk of bias taken into account in the interpretation and discussion**		1	1	1	1	1	2	1	1	1	1	1	2	1	1	1	1
**Satisfactory explanation for any heterogeneity**		4	1	1	4	2	4	1	1	1	1	4	2	2	1	1	1
**Assessment of presence and likely impact of publication bias**		1	1	1	1	1	1	1	1	1	1	1	2	1	1	1	1
**Conflicts of interest**		1	1	1	1	1	1	1	1	1	1	1	2	1	1	1	1

Legend: Criterion identified in the text, ___; criterion partially identified in the text, ___; unidentified criterion in the text, ___; and not applicable, ___.

**Table 5:** Antibiotic dosages evaluated for infection prevention in included studies of third-molar extractions.

Authors	Type of Antibiotics	Dosage of Antibiotics
Marcussen et al. [55]	Preoperative oral amoxicillin Preoperative penicillin V	single 2 g dose single 0.8 g dose
Milic et al. [59]	Preoperative oral amoxicillin	single 2 g dose
Falci et al. [51]	Postoperative amoxicillin/amoxicillin combined with clavulanate Preoperative metronidazole	750 mg/ 500 mg + 125 mg or 2000 mg + 125 mg 800 mg (duration of the therapy not provided)
Sologova et al. [61]	Amoxicillin combined with clavulanic acid at a ratio of 7:1	875 mg + 125 mg for 6 days
Wang et al. [52]	Amoxicillin and amoxicillin–clavulanic acid	1 g/2 g and 500 mg + 125 mg preop from 1h to 3 days before

## Data Availability

Data are deposited at the University of Genoa DISC and available upon request.
